# Supplementary material for: Robo-advisor acceptance: Do gender and generation matter?
Source: PLoS One. 2022 Jun 29;17(6):e0269454. doi: 10.1371/journal.pone.0269454 (PMC9242437; doi:10.1371/journal.pone.0269454)
Supplement: S1 Appendix — (DOCX) [file pone.0269454.s001.docx]

# Appendix A

Table A.1: Questionnaire (English): Section 1

| **Question** | **Answer** |
| --- | --- |
| 1. What gender do you recognize yourself in? | Female |
|  | Male |
|  | Other |
| 1. Your age in completed years | (age) |
| 1. Highest educational degree achieved so far | High School |
|  | Bachelor |
|  | Graduate Studies |
| 1. Which is your occupation in the University? | Student |
|  | Other |
| 1. What level of financial education do you think you have? | Low |
|  | Intermediate |
|  | High |
| 1. Do you think you are able to evaluate the financial offers received from specialized subjects? | No, I trust the proposals |
|  | No, I am not confident but I try to obtain independent information |
|  | Yes, I am usually able to evaluate financial offers |
| 1. Do you use the internet to gather information for making financial choices? | Often |
|  | Seldom |
|  | Never |
| 1. What is your investment horizon in years? | <1 |
|  | 3 |
|  | 5 |
|  | 10 |
|  | >10 |
|  | No |
|  |  |
| 1. Have you ever used a robo-advisor? | Yes |
|  | No |
|  |  |
| 1. Are you interested in robo-advice? | Yes |
|  | No |
| 1. Are you afraid of cyber risks which may be related to the use of robo-advice? | No, I trust the platform security |
|  | Yes, that’s why I am not interested |
|  | Yes, but I would use it anyway |
|  | I have no idea |

Table A.2. Questionnaire (English): Section 2, Likert-Scale Questions

| **I - Perceived Ease of Use (PEU)** | |
| --- | --- |
| I feel it would be easy for me to use the robo-advisor platform | PEU1 |
| I feel that the robo-advisor would be a clear and understandable tool to interact with | PEU2 |
| I feel it would be easy for me to become an expert in using the robo-advisor | PEU3 |
| I feel I would find the robo-advisor a flexible to interact with | PEU4 |
| It would be easy for me to get the robo-advisor to do what I want to | PEU5 |
| I feel I don't have enough experience to evaluate | PEU6 |
| **II - Perceived Usefulness (PU)** |  |
| Using the robo-advisor for my investments would enable me to achieve the desired results more quickly | PU1 |
| Using the robo-advisor would help me to do more efficient investment choices | PU2 |
| Using the robo-advisor would help me increase my returns | PU3 |
| **III - Attitude Towards Use (ATU)** |  |
| I believe that it is a good idea to use the robo-advisor | ATU1 |
| I like the idea of using the robo-advisor | ATU2 |
| I believe that using the robo-advisor is a wise choice | ATU3 |
| **IV - Behavioral Intention to Use (BIU)** |  |
| I intend to try to use a robo-advisor application within the year | BIU1 |
| Assuming I have access to the robo-advisor platforms, I intend to use them in the next few years | BIU2 |
| **V - Personal Investment Approach (PIA)** |  |
| I am very active in managing my investments | PIA1 |
| I'm fine with an algorithm making the decisions for me | PIA2 |
| I prefer that an algorithm or a human consultant interact with me according to my needs | PIA3 |

## Appendix B

We sum up here the estimation of the TAM path coefficients when estimated on the whole sample (214 observations). Parameters are obtained through the partial least squares method for structural equations (PLS-SEM) using SmartPLS software [1], and confidence intervals and t-test statistics to assess parameters’ significance are obtained by applying the bootstrap method [2, 3].

Several model specifications are considered: Model 0 includes only the basic TAM construct and the external variable PIA. Model 1 adds a control for education to the basic model and is proved to be non-significant, confirming an overall education homogeneity of the sample. Models 2 and 3 take into account generational cohorts, coded into a categorical variable with three outcomes in Model 2 or through dummy variables in Model 3. Some of the parameters estimates display significant values, suggesting generational cohort as a moderator for the TAM path links. Finally, Model 4 adds a dummy variable for female respondents to test for gender effects in the TAM model, and no significance is detected in interaction with the latent constructs.

Table B1. Path coefficients estimates for the entire dataset when suitable controls are included

|  | **Model 0** | **Model 1  Edu** | **Model 2 GenGroup** | **Model 3 Dummies for GenY and GenX^+^** | **Model 4 Female Dummy** |
| --- | --- | --- | --- | --- | --- |
| H1 PEU to PU | 0.432 | 0.431 | 0.421 | 0.422 | 0.432 |
| H2 PU to ATU | 0.695 | 0.688 | 0.682 | 0.691 | 0.691 |
| H3 PEU to ATU | 0.167 | 0.161 | 0.160 | 0.157 | 0.162 |
| H4 ATU to BIU | 0.703 | 0.700 | 0.689 | 0.686 | 0.691 |
| H5 PIA to PEU | 0.375 | 0.366 | 0.356 | 0.358 | 0.375 |
| H6 PIA to PU | 0.450 | 0.448 | 0.444 | 0.439 | 0.475 |
| EDU-PEU |  | *−0.074* |  |  |  |
| EDU-PU |  | *−0.018* |  |  |  |
| EDU-ATU |  | *−0.082* |  |  |  |
| EDU-BIU |  | *−0.019* |  |  |  |
| GEN_G-PEU |  |  | −0.139 |  |  |
| GEN_G-PU |  |  | *−0.076* |  |  |
| GEN_G-ATU |  |  | *−0.075* |  |  |
| GEN_G-BIU |  |  | *−0.058* |  |  |
| GEN_Y-PEU |  |  |  | *−0.080* |  |
| GEN_Y-PU |  |  |  | *0.004* |  |
| GEN_Y-ATU |  |  |  | −0.093 |  |
| GEN_Y-BIU |  |  |  | 0.122 |  |
| GEN_X^+^-PEU |  |  |  | −0.145 |  |
| GEN_X^+^-PU |  |  |  | *−0.085* |  |
| GEN_X^+^-ATU |  |  |  | *−0.073* |  |
| GEN_X^+^-BIU |  |  |  | *−0.076* |  |
| Female-PEU |  |  |  |  | *0.000* |
| Female-PU |  |  |  |  | *0.080* |
| Female-ATU |  |  |  |  | *−0.067* |
| Female-BIU |  |  |  |  | *−0.070* |

Note: EDU=education level; GEN_G=generation group (categorical); GEN_X^+^= Generation X^+^ dummy; GEN_Y=Generation Y dummy. Numbers in italics are non-significant at the 5% level.

In Table B2 we report the Akaike information criteria for Model 0, Model 2 and Model 3. The figures for Model 2 improve the basic model for all the constructs but the *behavioral intention*. The values for Model 3 are lower than those for the basic model, with the only exception of the *perceived usefulness*. Overall, a possible moderation effect is confirmed and deserves further investigation.

Table B2. Akaike information criterion for the basic model and considering the effect of generational groups

|  | **Model 0** | **Model 2 GenGroup** | **Model 3 Dummies for GenY and GenX^+^** |
| --- | --- | --- | --- |
| PEU | −29.494 | −32.256 | −30.332 |
| PU | −159.217 | −159.924 | −158.663 |
| ATU | −219.752 | −221.069 | −221.398 |
| BIU | −142.961 | −142.289 | −150.373 |

**Appendix C**

This Appendix collects all the outcomes for the multi group estimation of the TAM parameters.

Table C1 collects the value of some useful statistics to assess the validity of model constructs, such as the Composite Reliability (CR) and the Average Variance Extracted (AVE), across all considered sub-groups. The reliability threshold for the former statistics is 0.60 for experimental studies, whereas the latter should be over 0.50, see [4]. The results evidence a weak validity of the AVE for the PIA construct. Note that, by removing one of the items (questions) related to this construct both CR and AVE statistics increase above the desired levels, by leaving unchanged the overall qualitative results and conclusions on the path links and moderation effects. However, this comes at the expense of the interpretability of the PIA latent construct so we decided to keep all the measurement items for this factor.

Table C1. Composite reliability (CR) and average variance extracted (AVE)

|  | PEU | PU | ATU | BIU | PIA |
| --- | --- | --- | --- | --- | --- |
|  |  |  |  |  |  |
| Panel a (whole sample) | | | | | |
| CR | 0.866 | 0.951 | 0.967 | 0.943 | 0.665 |
| AVE | 0.617 | 0.867 | 0.906 | 0.892 | 0.414 |
| Panel b (sub-groups) | | | | | |
| Generation Z |  |  |  |  |  |
| CR | 0.843 | 0.949 | 0.960 | 0.930 | 0.618 |
| AVE | 0.571 | 0.860 | 0.888 | 0.870 | 0.396 |
| Generation Y |  |  |  |  |  |
| CR | 0.899 | 0.958 | 0.964 | 0.970 | 0.720 |
| AVE | 0.715 | 0.884 | 0.899 | 0.942 | 0.478 |
| Generation X+ |  |  |  |  |  |
| CR | 0.861 | 0.946 | 0.971 | 0.926 | 0.640 |
| AVE | 0.609 | 0.854 | 0.919 | 0.863 | 0.396 |
| Males |  |  |  |  |  |
| CR | 0.881 | 0.933 | 0.973 | 0.950 | 0.648 |
| AVE | 0.662 | 0.823 | 0.924 | 0.905 | 0.405 |
| Females |  |  |  |  |  |
| CR | 0.855 | 0.963 | 0.959 | 0.932 | 0.635 |
| AVE | 0.587 | 0.897 | 0.885 | 0.873 | 0.390 |

Note. ATU = attitude towards use; BIU = behavioural intention to use; PEU = perceived ease of use; PIA = personal investment approach; PU = perceived usefulness;

Table C2 collects parameter values and t-test p-values for path coefficients across the three generation cohorts.

Table C2. Path coefficients estimates and corresponding p-values for generational cohorts

| Hypothesis | | Gen X+ | | Gen Y | | Gen Z | |
| --- | --- | --- | --- | --- | --- | --- | --- |
|  |  | Path-coeff | p-value | Path-coeff | p-value | Path-coeff | p-value |
| H1 | PEU 🡪 PU | 0.263 | 0.029 | 0.587 | 0.000 | 0.566 | 0.000 |
| H2 | PU 🡪 ATU | 0.743 | 0.000 | 0.690 | 0.000 | 0.560 | 0.000 |
| H3 | PEU 🡪ATU | 0.067 | 0.541 | 0.229 | 0.078 | 0.298 | 0.001 |
| H4 | ATU 🡪 BIU | 0.700 | 0.000 | 0.757 | 0.000 | 0.673 | 0.000 |
| H5 | PIA 🡪 PEU | 0.187 | 0.242 | 0.422 | 0.001 | 0.509 | 0.000 |
| H7 | PIA 🡪 PU | 0.580 | 0.000 | 0.319 | 0.008 | 0.315 | 0.000 |

Note. ATU = attitude towards use; BIU = behavioural intention to use; PEU = perceived ease of use; PIA = personal investment approach; PU = perceived usefulness.

Table C3 collects parameter values and t-test p-values for path coefficients.

Table C3. Path coefficients estimates and corresponding p-values for gender groups

| Hypothesis | | Males | | Females | |
| --- | --- | --- | --- | --- | --- |
|  |  | Path  coefficient | p-value | Path  coefficient | p-value |
| H1 | PEU 🡪 PU | 0.448 | 0.000 | 0.424 | 0.000 |
| H2 | PU 🡪 ATU | 0.821 | 0.000 | 0.635 | 0.000 |
| H3 | PEU 🡪 ATU | 0.062 | 0.766 | 0.206 | 0.018 |
| H4 | ATU 🡪 BIU | 0.705 | 0.000 | 0.695 | 0.000 |
| H5 | PIA 🡪 PEU | 0.440 | 0.000 | 0.303 | 0.001 |
| H6 | PIA 🡪 PU | 0.495 | 0.000 | 0.429 | 0.000 |

Note. ATU = attitude towards use; BIU = behavioural intention to use; PEU = perceived ease of use; PIA = personal investment approach; PU = perceived usefulness.

To give an overall evaluation of the estimation results, we report in Table C4 the Adjusted R^2^ contribution of all constructs; values are reported across generational and gender groups and are within an acceptable range for this kind of analysis, the least contribution given by the *perceived ease of use*, especially for Generation X^+^ and women. Finally, Table C5 collects the correlation between latent constructs across all sub-groups.

Table C4. Adjusted R^2^ square statistics for the constructs, computed across sub-groups

|  | PEU | PU | ATU | BIU |
| --- | --- | --- | --- | --- |
| Generation Z |  |  |  |  |
| Adj. R^2^ | 0.252 | 0.593 | 0.637 | 0.447 |
| Generation Y |  |  |  |  |
| Adj. R^2^ | 0.157 | 0.585 | 0.744 | 0.563 |
| Generation X+ |  |  |  |  |
| Adj. R^2^ | 0.017 | 0.449 | 0.551 | 0.447 |
| Males |  |  |  |  |
| Adj. R^2^ | 0.187 | 0.630 | 0.708 | 0.459 |
| Females |  |  |  |  |
| Adj. R^2^ | 0.082 | 0.465 | 0.583 | 0.478 |

Note. ATU = attitude towards use; BIU = behavioural intention to use; PEU = perceived ease of use; PIA = personal investment approach; PU = perceived usefulness.

Table C5. Correlation coefficient for the constructs, computed across sub-groups.

| **Correlation** | **Whole Sample** | **Gen Z** | **Gen Y** | **Gen X^+^** | **Female** | **Male** |
| --- | --- | --- | --- | --- | --- | --- |
| **BIU - ATU** | 0.692 | 0.673 | 0.757 | 0.675 | 0.683 | 0.694 |
| **PEU- ATU** | 0.566 | 0.704 | 0.727 | 0.307 | 0.579 | 0.552 |
| **PEU - BIU** | 0.487 | 0.544 | 0.587 | 0.279 | 0.525 | 0.452 |
| **PIA - ATU** | 0.634 | 0.583 | 0.628 | 0.696 | 0.691 | 0.561 |
| **PIA - BIU** | 0.615 | 0.607 | 0.597 | 0.603 | 0.618 | 0.589 |
| **PIA - PEU** | 0.373 | 0.509 | 0.422 | 0.177 | 0.444 | 0.298 |
| **PU - ATU** | 0.787 | 0.776 | 0.855 | 0.750 | 0.845 | 0.749 |
| **PU - BIU** | 0.702 | 0.686 | 0.743 | 0.653 | 0.759 | 0.658 |
| **PU - PEU** | 0.599 | 0.726 | 0.722 | 0.367 | 0.666 | 0.551 |
| **PU - PIA** | 0.612 | 0.603 | 0.567 | 0.630 | 0.692 | 0.557 |

Note. ATU = attitude towards use; BIU = behavioural intention to use; PEU = perceived ease of use; PIA = personal investment approach; PU = perceived usefulness.

**References**

1. Ringle MC, Wende S, Becker JM. SmartPLS 3 (Software) Boenningstedt, Germany: SmartPLS; 2015.

2. Efron B. Better bootstrap confidence intervals. J Am Stat Assoc. 1987;82(397):171-85. https://doi.org/10.1080/01621459.1987.10478410.

3. Horowitz JL. The bootstrap. In: Heckman JJ, Leamer E, editors. Handbook of econometrics. Amsterdam, The Netherlands: Elsevier; 2001. p. 3159-228.

4. Kumar S, Goyal N. Evidence on rationality and behavioural biases in investment decision making. Qualitative Research in Financial Markets. 2016;8(4):270-87. https://doi.org/10.1108/QRFM-05-2016-0016.
