# Supplementary material for: Robo-advisor acceptance: Do gender and generation matter?
Source: PLoS One. 2022 Jun 29;17(6):e0269454. doi: 10.1371/journal.pone.0269454 (PMC9242437; doi:10.1371/journal.pone.0269454)
Supplement: Attachment — Submitted filename: AnswersReviewer2 30092021.pdf [file pone.0269454.s003.pdf]

Answers to Reviewer 2

Dear Reviewer, thank you for your time and interest in the paper. We are very grateful for the comments and suggestions you provided which truly helped us improving the manuscript. In what follows, after a description of general changes, we give a detailed answer to your specific comments and describe (in italics) how your concerns or suggestions were addressed in the revised manuscript

General description of changes

1) *The title has changed to* "Robo-advisor acceptance: do gender and generation matter?"

   Indeed, we have considered gender in a more extensive way, as suggested by one reviewer, and the Journal has required a shorter and impactful title.

2) *The names of generations cohorts have been changed throughout the paper in Generation Z (former Digital Natives), Generation Y (former Millennials) and Generation X^{+} (former "older generations" including Generation X and Baby Boomers). We haven't highlighted this writing change in the "tracked changes" version where the form and meaning of the sentence was not modified. The actual segmentation by age is described in line 152 of the current version.*

3) *As proposed by the Editor, we referred to related literature on technology adoption within generation cohorts in order motivate better our research question; in addition, following the suggestion of one reviewer we performed a preliminary analysis on the entire sample with the inclusion of gender and age as a control variable, the latter being coded in several ways (lines 219-256 and Tables therein) proving that age does matters in robo-advice adoption.*

4) *We followed the suggestion of the Academic editor to include technical details on the PLS-SEM model estimation following* Hair et al. (2019). *Specifically, we have added a better description of the TAM model, within the PLS-SEM framework (lines 77-81,105-119) and a set of procedures to pre-validate model constructs (lines 185-208 and Tables therein). Moreover, we have applied the MICOM procedure prior to the Multi Group analysis to guarantee that the same constructs are valid for the considered subgroups (209-217).*

Below the answers to your specific comments (in italics).

Specific comments

RESEARCH QUESTIONS AND CONCEPTUALIZATION

1. The motivation and relevance of the research questions (the moderating effect of age) should be strengthened. Currently, the authors mention they "believe" age may be important. While the (moderating) effects of age have been investigated in several technology-related domains before, why is age important in the specific context of robo-advice, in particular financial robo-advice? Lourenço, Dellaert, and Donkers (2020), for instance, have included age as a control in their recent study of robo-advice acceptance.

We have strengthened the motivation by referring to few other papers where this distinction is important and relevant for model design in technology adoption context such as Mahmoud et al 2021. In fact, the relationship is controverted in robo-advising literature; while Chen et al. (2019) find ***that age (considered as a control variable) is insignificant with respect to trust on robo-advisory,*** *Lourenço et al. (2020) find a significant (negative) impact of age,* ***all else equal, on the satisfaction in using automated tools for financial advice.*** *Hence, understanding whether there are (or not) significant differences in our context may help the Robo-advising platforms to target generations differently (or not).*

In addition, we run the PLS-SEM algorithm for the whole sample with the inclusion of a control variable for Age with three different approaches:

1) *Continuous valued CV=Age*
2) *Categorical Variable CV=GenGroup values=1,2,3 according to our choice of generational cohorts.*
3) *Two Dummies CVs for Generation Y (former Millennials) and Generation X^+ (former Generation X and Baby Boomers) with Generation Z (former Digital Natives) as reference group.*

The outcomes evidence that age/generation are relevant for the topic under investigations. The results are reported in lines 219-256 and Tables therein.

2. And then the authors discuss why there could be differences between the two youngest groups but not between those and the oldest groups.

We removed a sentence which was somehow misleading. We also added references of papers addressing such generational changes (Ameen et al. (2021), Mahmoud et al. (2021) and added some comments in the discussion.

3. The authors should also discuss, even if briefly, the differences between age-, cohort-, and period-effects and the challenges to disentangle the three. In so doing, they should clarify that they recode age into generational cohorts out of convenience, but they do not intend estimate the effects of age vs. cohort (or age vs. period nor cohort vs. period).

To enhance the motivation for our study and address your concerns here and also answer your comments in Methodology (point 4) we ran the PLS-SEM algorithm for the whole sample with the inclusion of a control variable for Age with three different approaches (CV=Age(continuous), CV=generational cohort (categorical) or CV1=Dummie for GenX vs Z, CV2=Dummy for GenY vs GenZ) and provided some comments on the results.

4. The hypotheses, which could perhaps be re-located to section 1, should be formulated in reference to the moderating effect of age and the differences across cohorts. The current hypotheses refer to the original TAM model, which have been tested countless times before.

We contextualized the hypotheses within the robo-advisory framework rather than only TAM acronyms. Of course, the TAM model has been estimated countless times but on other topics or under different assumptions and the reader nay not be familiar with TAM assumption. Anyway, we followed your suggestion by pointing out that we test the assumptions plus the possible moderation of age/generation (and gender).

5. In the introduction, the authors could add on p. 3, line 34 the work by Baker and Dellaert (2017).

Thank you for this reference, we have included it.

6. While the authors also emphasize, and analyze in detail, the effect of gender, the introduction and conceptualization are silent about it. As such, mentioning on page 6, lines 98-100, that the purpose of the study is two-fold comes as a surprise, to say the least.

We agree with you that we didn't emphasize this part of the research. Since we found no significant difference in gender, we focused on generational differences. However, after your comment and a second thought, we realized that, exactly because they are not aligned with previous literature (when educational level is not homogeneous), they may also be relevant to evidence that education may boost females' acceptance of fintech solutions. Hence, we have extended our conceptualization on possible gender differences and detailed the results (lines 284-300).

METHODOLOGY AND RESULTS

1. The authors do not explain how they have recoded age into generational cohorts, and what past sources have they followed to do that. That is, what birth years (current age) were considered to classify individuals into the four different cohorts?

We have overall 3 (not 4) groups in the analysis. To avoid confusion, we renamed the generational cohorts in the revised version of the paper and specified the age ranges in terms of birth year (see general changes).

2. The authors should also be consistent in their use of generational labels. For instance, sometimes they refer to young individuals as Millennials and sometimes as Generation Z, or

Digital Natives and Generation Y. Also, are Millennials Gen. Z or Gen. Y? More importantly, 'Digital Natives' may be misleading, as Millennials are often considered Digital Natives too.

We changed the terminology to be consistent in the whole paper (see general changes).

3. The authors should report the fit measures typically used when estimating structural equation models, and show in particular that a model with age (and age cohorts) outperforms in terms of fit a model without age. They could use a likelihood ratio test.

We have reported the adjusted R^2 but we refrain from using other model fit indicators since they need to be taken with caution as there are noy yet established research and rules of comparison. Besides, we cannot compare the whole dataset fitting results with the subgroups, which have a different number of observations.

In order to establish the general need to take generational cohorts into account we compared model AIC values for the whole sample case whether or not age or generational cohorts have been included in the model (Table 7 in the revised manuscript). The results show that taking into account age or cohorts improve the above statistics.

4. Do the results change if instead of a multigroup analysis the authors estimate a model with the entire sample but include the moderating variables (3 dummy variables, one for each of the three cohorts that compare to the reference cohort, Gen. Z)? While comparing such model, the authors should not only include the moderating variables but also the cohort dummies as main effects.

We thank the reviewer for suggesting this alternative approach. Indeed, moderation can be performed alternatively by using interactions or MG analysis. We adopted the second strategy because we wanted to investigate possible changes in all the path relationships considered in the theoretical base model rather than in one specific path.

Anyway, we did run the suggested model (to answer your question) with two dummies (Gen Y and GenX+) when GenZ is taken as the reference group: outcomes confirm that interactions with GenX+ are actually significant prom PIA to PEU and PIA to PU (Hypo 5 and 6 in the original manuscript) and from PEU to PU (Hypo 1). Results have been reported in the new version (lines 337-344).

5. What is the number of respondents who fall into the two oldest cohorts, Gen. X and Baby Boomers? In Table 1, we can only see that, jointly, there are 70 respondents in the 'other' age group.

We did not divide the group of "older generations" since the distinction is not relevant for the paper. In fact, see comments 1-2 above, we have overall 3 (not 4) groups in the analysis: GenZ (former Digital Natives), Gen Y (former Millennials) and Gen X+ (former "older generations" including Generation X and Baby Boomers).

6. What is the response rate, i.e. the ratio of respondents to the number of individuals who received the link?

It is difficult to estimate the actual ratio between who responded and who received the link. We had around 23000 students enrolled in 2019 and around 2000 faculty and staff. If we consider these numbers as our potential respondents the response rate is around 1.5% (with completed questionnaire in 0.9 % of the overall "emails").

However, the email with the link to the questionnaire had been sent to students' official mail (@studenti.unipg.it) which is seldom checked by students, so that lately the University has updated all the communication systems in order to make registration with personal email possible (@gmail, @cloud, @microsoft or any other). In the paper we added a footnote where we distinguished students and staff respondent's percentages, which are around 0.45% and 5.00-6.00% respectively.

CONCLUSIONS

The authors should discuss, even if briefly, the limitations of their study, namely (i) the fact that it is only based on self-reported measures (it is not a behavioral study where respondents get to evaluate an actual financial robo-advice algorithm or interface), and (ii) their sample is an Italian sample of highly educated individuals.

Thanks for this remark, we added the above observations on the limits of our study at the end of the discussion section .

Reported typos

Reported typos have been corrected

References:

Ameen, N., Hosany, S., & Tarhini, A. (2021). Consumer interaction with cutting-edge technologies: Implications for future research. *Computers in Human Behavior*, *120*, 106761

Baker, Tom, and Benedict Dellaert. "Regulating robo advice across the financial services industry." Iowa L. Rev. 103 (2017): 713.

Lourenço, C. J. S., Dellaert, B. G. C., & Donkers, B. (2020). Whose algorithm says so: The relationships between type of firm, perceptions of trust and expertise, and the acceptance of financial robo-advice. Journal of Interactive Marketing, 49, 107–124.

Mahmoud, A.B., Hack-Polay, D., Grigoriou, N. *et al.* (2021) A generational investigation and sentiment and emotion analyses of female fashion brand users on Instagram in Sub-Saharan Africa. *J Brand Manag* **28,** 526–544 (2021). https://doi.org/10.1057/s41262-021-00244-8:
