# Supplementary material for: Robo-advisor acceptance: Do gender and generation matter?
Source: PLoS One. 2022 Jun 29;17(6):e0269454. doi: 10.1371/journal.pone.0269454 (PMC9242437; doi:10.1371/journal.pone.0269454)
Supplement: Attachment — Submitted filename: AnswersReviewer3.pdf [file pone.0269454.s004.pdf]

Manuscript: Robo-advisor acceptance: do gender and generation matter?

Answers to Reviewer 3

Dear Reviewer, thank you very much for going through our paper and for your suggestions, that we were happy to follow.

Note that the paper has been extensively revised to focus on the moderation effect of generations and genders and both the preliminary results on the whole sample and the model assessment validation have been moved to the Appendix, in order to accomplish the request of another reviewer.

We believe that the current version has gained in clarity, effectiveness and readability.

In italics we give here below the answers to your specific comments, which are also reported for your convenience.

1. While the gender comparison did not produce notable differences, the paper does describe some minor gender gaps that may still require attention. I suggest that the authors note finding minor gender differences and stating their nature in the abstract, as just saying "we do not find significant differences between male and female" may lead readers to think that no differences were found.

Indeed, we find some differences, but they are not statistically significant. We agree that our sentence in the abstract can be confusing and we have rephrased it to avoid any misunderstanding. Thank you for pointing this out.

2. Overall, it is interesting - refreshing yet surprising - that no significant gender gaps were found, as much of research on new technology adoption in the recent years show that men are more accepting of new technologies than women in various domains. Is there something that's different about the nature of robo-advisors that may contribute to the lack of differences? Could it be a result of the sample composition, e.g., did males and females in the sample share comparable characteristics, or were there characteristics of the sample that may have contributed to the muted gender effects?

You are right, the results are a consequence of the special characteristics of the sample (the high education of the respondents), that, in our opinion, contribute to levelling the gender gap. We have better highlighted this aspect of the research.

3. The very last paragraph stating limitations of the study needs to be elaborated much further. The sample composition poses additional challenges and limitations than what the authors have mentioned, as there are additional characteristics that make the sample far from representative. A deeper discussion of the sample limitations, and how these may have contributed to the

findings need to be discussed. Additionally, it would be helpful to see examples and suggestions around the "behavioral studies" proposed to improve the objectivity and validity of the research.

We have elaborated more on the limitations of the study in the concluding section.

Regarding the possibility to carry on an analysis without the limit of self-reported data, a possible approach is to perform virtual experiments on (virtual) portfolio management where respondents are guided through specific choices, also involving robo-advisory possible applications (behavioral finance approach). Alternatively, one can possibly request real data from specific financial platforms supporting the use of robo-advice, such as in Bianchi-Brière (2021), to analyze how investors manage their portfolio in reality. Of course, these are sensible data and not easy to obtain. Anyway, since we realized that such a comment did not add much to the discussion of our results, we deleted the reference to behavioral studies.

4. While the paper is written in standard language and does not have major grammatical errors, use of non-academic language and subjectively toned sentences are noticeable throughout the discussions which seem unfit for a research manuscript.

The revised paper has undergone a full proofreading process with a professional. We hope that any inappropriate language has been removed from the text.
