# Supplementary material for: Robo-advisor acceptance: Do gender and generation matter?
Source: PLoS One. 2022 Jun 29;17(6):e0269454. doi: 10.1371/journal.pone.0269454 (PMC9242437; doi:10.1371/journal.pone.0269454)
Supplement: Attachment — Submitted filename: AnswerReviewerFinal.pdf [file pone.0269454.s005.pdf]

Answer to Reviewer report on “Robo-advisor acceptance: do gender and generation matter?”
Manuscript PONE D-21-14526R2

We would like to thank the reviewer once again for his/her insightful comments which made us reflect on some possible concerns in our research design and come out with more convincing motivations for our setting. Precisely, a main issue is raised relative to the choice of reporting the results by grouping respondents according to generation cohorts. As the reviewer pointed out, this is done in practice by splitting the sample according to the age variable at the time of the survey, and he/she is concerned that our findings may be age-triggered rather that cohort-related. This is an accountable concern and the definition of generational cohorts has been also questioned by Rudolph and Zacher (2016).
However, most of the papers addressing potential differences across generational cohorts, no matter the application of interest, do not discuss explicitly the social and psychological characteristics necessary for an age group to suitably represent a generational cohort, but rather rely on already existing classifications (based on the birth-date).

The age-groups in our paper are based specifically on the generation classification given by Williams et al. (2010) but it is consistent with other accredited and used classifications which are cited in the new version of the paper and added to the references.

For example, Turner (2015) suggests that Baby boomers are those born in the time range 1946-64 Gen X within the period 1965-1977, GenY are born from 1977 to 1993 and **Gen Z are born in 1994-2005**; Cilliers, E. J. (2017) considers the following classification: Baby boomers are those born in 1945-1965, Gen X are born in 1965-1979, Gen Y in 1980-1994and **Gen Z after 1994**.
We are aware that some papers suggest that Generation Z may start later on (1998 or 2000), for example Mahmoud et al. (2021). Due to our choice of having a highly educated sample where younger respondents are university students (who are necessarily over 18 in the Italian school system), we decided to consider the larger classification of Generation Z, starting from 1994.

It is worth noticing that in lines 146-148 of the submitted manuscript (R2), where we write *“Respondent ages range from 18 to 67 years and are grouped by generational cohort: Generation Z (born 1994–2012; 47%), Y (born 1980–93; 19.6%) and X⁺ (born before 1980; 32.7%)”*, the year ranges in parenthesis corresponds to the definition of the cohorts in the literature, not the birth dates for our sample. Indeed, as reported in the manuscript, the survey was conducted at the end of 2019 the youngest students were born no later than year 2001, although the range describing this generation goes up to 2012.

In fact, in this and similar studies, it is difficult to disentangle age and cohort effects. For example, imagine that just two groups are considered, younger (18 to 40 years old) and older (>=41 y.o.), and assume they are associated to two different generations (Younger to Gen B and Older to Gen A). In order to disentangle a generational cohort effect from an age effect one should in principle wait up to 20 years to repeat the same analysis when most respondents of Gen B aged over 41. If the results of the future survey for Gen B will be the same of the of the current survey, then we can say that the findings are associated to the generation cohort; conversely, if the future results for Gen B will be in line with current results for Gen A, then we can say that they are age-related.

Nevertheless, some research questions are driven by the interest of investigating the change of habits which come with renewal of generations rather than aging, though we agree that the definition of a generation cohort can be imprecise and that the results should be analyzed with caution. We include our research among these, and we believe that when it comes to attitude to new technologies, especially concerning financial decision and planning, the behavior of respondents can indeed change with a generation “renewal”.

Of course, we could simply relate our results to age-groups but the analysis of differences across generational cohorts is an important line of research but, despite the intrinsic limitations in the definition of the generation groups, this change is what we would like to investigate in our research.

With this limitation, we still believe that our contribution fits well within the generational cohorts literature stream and can give some insights about different attitudes towards fintech and specifically, the robo-advisory service which we deal with in the submitted manuscript.

Hence, we decided to stick with the generation groups while better acknowledging in the paper, both in the sample description (Section 3) and in the discussion (Section 5), the intrinsic limitations in generational cohorts' studies.

We answer below to specific points raised by the reviewer (original comments are reported in italics):

1) *I'm pleased that the authors have considered seriously several suggestions to improve their work. Thus, the research problem and specific research questions are now clearer, and as a result so is its contribution. Also, the conceptualization is tighter, namely in respect to the main moderating variable being analyzed, i.e. age, and there are now explicit hypotheses being tested. The paper no longer oversells nor speculates, which makes it more rigorous and increases its validity -- hopefully translating into a larger impact over time*.

We are very happy that you have appreciated our effort to make the research question and method clearer and we would like to thank your guidance which helped substantially for us to achieve this goal.

2) *Having said that, I would like to stress that a generational or cohort analysis is not what the authors do in this paper. Apart from the usually loose definition of generational groups that the authors also mention, the authors' generational groupings do not support their definition. As the authors state, "generations have a different culture" but it is hard to accept that individuals born in say 1995 have the same culture, i.e., belong to the same cohort, as individuals born in say 2010 (some fifteen years later), as it is implied by their grouping into Generation Z.*

We have clarified our viewpoint both above and in the paper, hopefully in a more convincing way. Anyway, note that the respondents in our research were born no later that 2001 (the range in parenthesis is the "official" range for birth dates of GenZ).

3) *It is even harder to accept that individuals born in say 1979 have the same culture, i.e., belong to the same cohort, as individuals born in say 1960 (some nineteen years before), as it is implied by their grouping into Generation X+.*

   Indeed, the last group is the largest in terms of age because we have grouped GenX and Baby Boomers and, giving credit to your concern, we ran the whole analysis by further splitting the GenX+ in GenX and Baby Boomers with 39 and 31 individuals, respectively. The outcomes evidence no significant differences between the path coefficients of the two sub-groups, though some of the GenX+ behaviours are exacerbated (for example the path coefficients for H3 (PEU to ATU) are 0.15 (p=0.274) and 0.009 (p=0.96) for Gen X and Baby Boomers respectively whereas this link coefficient is 0.067 (p=0.541), when the two cohorts are aggregated in a single group. Since the overall contribution is not affected by the above split, we decided not to change the whole paper construction, and just acknowledge this further investigation.

4) I insist this is a fundamental issue in the paper. And I would like to again explain that what the authors really do is to discretize (group, if you will) the age variable they have included in their survey. Apart from issues regarding the loss of information when discretizing a continuous variable that I've discussed in the first round, please note that such discretization, which is arbitrary, cannot by any means be seen as a cohort analysis. Actually, there are no "generation"-like arguments from say sociology in the conceptualization section nor in the concluding sections. The analysis really is a typical age moderation analysis and there's nothing wrong with that.

You are right, there are no generation-like arguments; honestly, we are not sociologists and our contribution is not at all the classification of age-groups in generational cohorts. Instead, we rely on the existing literature on the matter, where the generation cohort classification (given by birth date) is supported in terms of sociological and psychological characteristics.

5) Lines 279 to 285, which by the way are the only ones in the paper offering some rational for why the age dimension may be interesting to look at as a moderator for robo-advice acceptance, are "age"-like arguments rather than "cohort"-like.

Based on our comments to you, we added some cohort-like arguments to our research conceptualization (l. 152-156).

6) *Therefore, I would strongly recommend the authors to remove all "generation"-like terminology in the paper (including in the title and in the abstract) and instead stick with the "age"-like terminology.*

Lines 146 to 148 can then be re-written as follows: "Respondent ages range from 18 to 67 years. To test our moderation hypotheses, we grouped respondents by birth year as follows: Younger (born...), Middle (born...), Older (born...).

As stated in the general comments we think that, despite the intrinsic limitation in the definition of a generation cohort, our contribution still fits in this line of research.

7) Please note that if there are respondents who were born in 2012 (and that you labeled Generation Z), then in 2019 by the time you have collected the data, they were 7 years old. You mention that the youngest respondents were 18, however.

As noticed in the manuscript, the respondents in the sample were born no later than in 2001 and the year 2012 is only mentioned as the extreme of the birth dates defining Generation Z in cited literature.

8) *Methodologically, please note that when reporting structural equation model results it is customary to report a correlation matrix with all variables included in the estimation as well as SEM fit indices, e.g., chi-square test, CFI, RMSEA.*

We added a Table to the Appendix reporting the correlation between the model constructs in the whole sample and the subgroups. As for the fit indices, we refrained from reporting them, as suggested in Hair et al. (2019), since their use and interpretation in PLS is still controversial.

9) *Minor comments:*
Table 2 can (should) be reduced in size.

Figures 1 and 2 should have a legend stating that the figures in parentheses are p-values.

We have added a sentence in the text to describe the quantities in the figures (l. 210-211). Anyway, we will agree with the editorial office to best way for tables and figures to fit the journal's requests.

References

Cilliers, E. J. (2017). The challenge of teaching generation Z. *PEOPLE: International Journal of Social Sciences*, *3*(1).

Hair JF, Risher JJ, Sarstedt M, Ringle CM (2019). When to use and how to report the results of PLS-SEM. *European Business Review*, 31(1).

Mahmoud AB, Hack-Polay D, Grigoriou N, Mohr I, Fuxman L. (2021) A generational investigation and sentiment and emotion analyses of female fashion brand users on Instagram in Sub-Saharan Africa. *Journal of Brand Management 28(5)*.

Rudolph CW, Zacher H. (2016). Considering generations from a lifespan developmental perspective. *Work Aging Retire* 3(2).

Turner, A. (2015). Generation Z: Technology and social interest. *The journal of individual Psychology*, *71*(2).

Williams, K. C., Page, R. A., Petrosky, A. R., & Hernandez, E. H. (2010). Multi-generational marketing: Descriptions, characteristics, lifestyles, and attitudes. *The Journal of Applied Business and Economics*, *11*(2).
